# Utility of Preoperative Distractive Stress Radiograph for Beginners to Extent of Medial Release in Total Knee Arthroplasty

**DOI:** 10.4055/cios.2009.1.2.110

**Published:** 2009-05-30

**Authors:** Jae Ang Sim, Ji Hoon Kwak, Sang Hoon Yang, Sung Hoon Moon, Beom Koo Lee, Joon Yub Kim

**Affiliations:** Department of Orthopaedic Surgery, Gil Medical Center, Gachon University, Incheon, Korea.

**Keywords:** Total knee arthroplasty, Femoro-tibial angle, Medial release, Distractive stress radiograph

## Abstract

**Background:**

This study evaluated the preoperative distractive stress radiographs in order to quantify and predict the extent of medial release according to the degree of varus deformity in primary total knee arthroplasty.

**Methods:**

We evaluated 120 varus, osteoarthritic knee joints (75 patients). The association of the angle on the distractive stress radiograph with extent of medial release was analyzed. The extent of medial release was classified into the following 4 groups according to the stage: release of the deep medial collateral ligament (group 1), release of the posterior oblique ligament and/or semimembranous tendon (group 2), release of the posterior capsule (group 3) and release of the superficial medial collateral ligament (group 4).

**Results:**

The mean femorotibial angle on the preoperative distractive stress radiograph was valgus 2.4° (group 1), valgus 0.8° (group 2), varus 2.1° (group 3) and varus 2.7° (group 4). The extent of medial release increased with increasing degree of varus deformity seen on the preoperative distractive stress radiograph.

**Conclusions:**

The preoperative distractive stress radiograph was useful for predicting the extent of medial release when performing primary total knee arthroplaty.

Well-balanced soft tissue is essential for achieving a good result when performing total knee arthroplasty. Soft tissue release should be precisely carried out step by step.

The preoperative planning is critical for ensuring a good operation. The preoperative radiographic distractive view can be used to predict the ligament balance. This study evaluated the preoperative distractive stress radiographs in order to quantify and predict the extent of performing medial release according to the degree of varus deformity in primary total knee arthroplasty.

## METHODS

We reviewed 120 joints in 75 patients who underwent primary posterior cruciate ligament substitution (PS) total knee arthroplasty from August, 2004 to December, 2006. The mean age of the patients was 66.5 years (range, 48 to 79 years). One hundred eleven knees were in 68 female patients and nine knees were in 7 male patients. All the patients had osteoarthritis with a varus deformity.

**Fig. 1:**
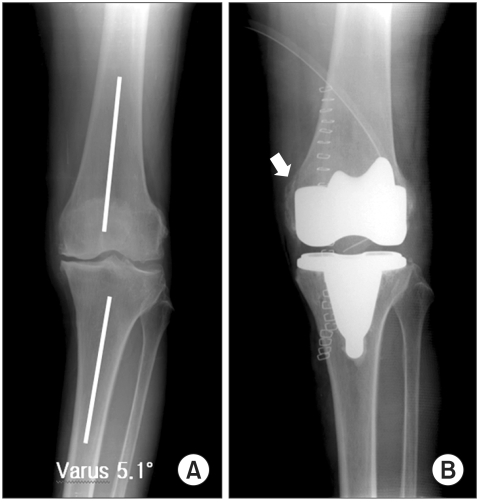
The femorotibial angle on the preoperative distractive stress radiograph showed varus 5.1° (A). Stage IV release with a medial epicondylar osteotomy was needed for proper ligament balance (B).

The preoperative distractive stress radiographs and the whole extremity radiographs were studied. The distractive stress radiograph was taken with the patient in the supine position. The examiner manually pulled the patient's legs into neutral rotation (Fig. 1). In all cases, one observer preoperatively measured the femorotibial angle on the whole extremity radiograph and the distractive stress radiograph, and the same surgeon performed medial release during total knee arthroplasty in order to decrease the interobserver variation. Patients for whom a true anteroposterior distractive stress radiograph could not be obtained due to flexion contracture and femoral deformity such as bowing and malunion were excluded because the distractive stress knee AP radiograph could not reflect the femorotibial angle of the whole extremity radiograph.

Medial release was performed after cutting bone perpendicular to the mechanical axis and resecting the posterior cruciate ligament (PCL) and the osteophytes on the medial compartment. The extent of medial release was classified into the following 4 groups according to stage: (1) release of the deep medial collateral ligament (group 1), (2) release of the posterior oblique ligament and/or semimembranous (SM) tendon (group 2), (3) release of the posterior capsule (group 3) and (4) release of the superficial medial collateral ligament (group 4). Group 4 included the complete distal release of the medial collateral ligament (MCL) and medial epicondylar osteotomy as proposed by Engh and Ammeen1) Medial epicondylar osteotomy was performed by removing the bone fragment from the medial femoral condyle by an osteotome while maintaining the continuity of the medial collateral ligament origin and the adductor magnus tendon insertion. Lamina spreaders that were situated parallel to the cut bone were placed on the medial and lateral femorotibial joint space after bone cutting.

Distraction was carried out manually to obtain a firm resistance. The extension and flexion gaps, both medially and laterally, were measured using calipers. The gap measurement was performed in full extension and then in 90° flexion. Release was performed step by step while measuring the extension gap medially and laterally. Proper gap balancing was determined as the surfaces of the cut bone were parallel. The association between the angle on the distractive stress radiograph and the extent of medial release was analyzed.

All statistical analyses were performed using the SPSS ver. 8.20 (SPSS Inc., Chicago, USA). MANOVA tests were used to determine the relationship between the femorotibial angle and the extent of medial release, and the groups were compared by Tukey's test. A *p* value < 0.05 was considered significant.

## RESULTS

Of the 120 cases for which medial release was performed, 30 (25.0%), 41 (34.2%), 20 (16.7%), and 29 (24.2%) cases were in group 1, 2, 3, and 4, respectively. Group 4 included 3 cases with complete release of the distal superficial MCL and 26 cases with a medial epicondylar osteotomy. After medial release, the difference between the medial and lateral gaps in flexion and extension was 0.1 mm (range, 0 to 1 mm) and 0.1 mm (range, 0 to 1 mm), respectively. The difference between the flexion and extension gaps was 0.6 mm (range, 0 to 1.5 mm). There were no significant difference (*p* > 0.05).

**Fig. 2:**
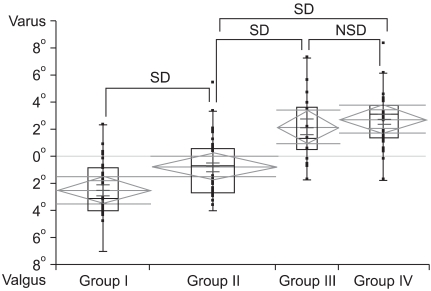
The femorotibial angles on the preoperative distractive stress radiograph. SD: Significant difference, NSD: No significant difference.

The mean femorotibial angle on the preoperative distractive stress radiograph for groups 1, 2, 3, and 4 was valgus 2.4° (valgus 6.9° to varus 2.3°), valgus 0.8° (valgus 4.0° to varus 5.4°), varus 2.1° (valgus 1.7° to varus 7.3°) and varus 2.7° (valgus 1.8° to varus 8.3°), respectively. The extent of medial release increased significantly with an increasing varus deformity seen on the preoperative distractive stress radiograph (*p*M: MANOVA *p* value, *p*M < 0.001). The femorotibial angles between groups 1 and 2, groups 2 and 3, and groups 2 and 4 were significantly different, but there was no significant difference between groups 3 and 4 (*p*TG: Tukey Grouping *p*-value, *p*TG12 = 0.0015, *p*TG23 < 0.001, *p*TG24 < 0.001, *p*TG34 = 0.3598) (Fig. 2).

## DISCUSSION

Even though intraoperative assessment of the ligament tension is most important for the successful outcome of total knee arthroplasty, preoperative assessment of the ligament tension is also important2) not only to recognize the quality of soft tissue, but also to make a preoperative assessment of such factors as flexion contracture and varus and valgus deformity. Mihalko and Krackow3) reported that the examination of the joint prior to exposure, with distractive tension and varus and valgus applied stress, can alert the surgeon to the need for a medial or lateral soft tissue release during revision total knee arthroplasty. We could conduct the operation more precisely and easily by meticulously evaluating the preoperative distractive stress radiograph and predicting the needed extent of medial release.

Some authors have focused on quantitatively measuring the relationship between the different soft tissue release techniques and their influence on correcting the deformity.^4^-6) They reported that there is no precise relationship between the amount of soft tissue release and the gap opening. However, they have confirmed a significant increase in correcting the deformity with using stepwise release sequences. In this study, the extent of medial release increased with the increasing degree of varus deformity seen on the distractive stress radiograph. On comparison of the groups, we could predict the extent of medial release in groups 1 and 2, groups 2 and 3 and groups 2 and 4, but the femorotibial angles between groups 3 and 4 showed no significant difference.

During total knee arthroplasty, achieving anatomic limb alignment and proper soft tissue balance are very important for preventing a destructive load on the joint and to create a stable joint over the full range of motion.^4^,^7^,8) The latter is one of the main factors that are critical to the longevity of a total knee arthroplasty prosthesis.

Many different procedures for gaining proper soft tissue balance have been reported.^4^,^7^,8) These include distraction testing methods with using tension jigs, laminar spreaders and spacer blocks or in-line traction to assess the joint gap after both the tibial and femoral cuts have been made.^2^,^9^,10) Another method for evaluating ligament balance is using trial components to fill the gaps and this is followed by the application of varus and valgus stresses.^11^,12) These techniques are useful for testing the ligament balance intraoperatively during the performance of total knee arthroplasty.

The optimal procedure for medial release is still controversial. Matsueda et al.5) recommended the following 8 sequential steps for medial release: anteromedial tibial sleeve released 2 cm below the joint line, release of the posteromedial capsule and tibial attachment of the SM, anteromedial tibial sleeve released 4 cm below the medial joint line, anteromedial tibial sleeve released 6 cm below the medial joint line, anteromedial tibial sleeve released 8 cm below the medial joint line, MCL released from the femoral condyle, release of half of the PCL and release of the entire PCL. Yagishita et al.4) sequentially performed release of the medial and posteromedial capsule, including the deep MCL and the SM, resection of the medial osteophytes, release of the tibial insertion of the MCL, a gradual cut of the MCL and partial release of the PCL. Bottros et al.2) sequentially performed removal of osteophytes, release of the pes anserine complex, the deep MCL and the posteromedial capsule complex, resection of the PCL and release of the superficial MCL. In this series, release of the deep MCL, the posterior oblique ligament and/or the SM, the posterior capsule and the superficial MCL were performed sequentially. The patients were classified into 4 groups according to the extent of medial release. A simple knee AP radiograph can reflect the whole extremity, but not the knee with a bony deformity.^13^,14) Therefore, in order to evaluate the femoral anatomic axis in a knee with deformity, a line was drawn connecting the midpoint of the shaft just inferior to the level of the lesser trochanter and the midpoint of the shaft 10 cm proximal to the knee joint.15) The cases with flexion contracture or femoral deformity were excluded because we could not obtain a true AP distractive stress radiograph for the cases with flexion contracture, and the anatomical axis observed on the simple knee AP radiograph could not reflect the true anatomical axis in the deformed knee. A whole extremity radiograph should be taken if the distractive stress radiograph of a deformed extremity is evaluated.

In conclusion, the extent of medial release can be quantified according to the degree of the femorotibial angle observed on the preoperative true AP knee radiograph in distraction.
